# Frontline perspectives on barriers to care for patients with California Medicaid: a qualitative study

**DOI:** 10.1186/s12939-024-02174-8

**Published:** 2024-05-22

**Authors:** Jessica Faiz, Mariah Blegen, Vanessa Nuñez, Daniel Gonzalez, Daniel C. Stokes, Kevin Truong, Gery Ryan, Medell Briggs-Malonson, Katherine L. Kahn

**Affiliations:** 1 National Clinician Scholars Program, VA Greater Los Angeles Healthcare System and UCLA, 1100 Glendon Ave., Ste. 1100, 90024 Los Angeles, CA USA; 2 Department of Surgery, David Geffen School of Medicine at UCLA, 10833 Le Conte Ave., 72-227 CHS, 90025 Los Angeles, CA USA; 3 David Geffen School of Medicine at UCLA, 757 Westwood Plaza, Ste. 7419, 90095 Los Angeles, CA USA; 4 Department of Medicine, David Geffen School of Medicine at UCLA, 757 Westwood Plaza, Ste. 7419, 90095 Los Angeles, CA USA; 5 Department of Health Systems Science, Kaiser Permanente Bernard J. Tyson School of Medicine, 100 S Los Robles Ave, Ste. 300, 91101 Pasadena, CA USA; 6 Department of Emergency Medicine, David Geffen School of Medicine, UCLA, 757 Westwood Plaza, Ste. 1320, 90024 Los Angeles, CA USA; 7 RAND Health, RAND Corporation, 1776 Main St., 90401 Santa Monica, CA USA

**Keywords:** Medicaid, Managed care, Access to care, Systems of care, Ambulatory care, Health equity, Social drivers of health

## Abstract

**Background:**

While insurance is integral for accessing healthcare in the US, coverage alone may not ensure access, especially for those publicly insured. Access barriers for Medicaid-insured patients are rooted in social drivers of health, insurance complexities in the setting of managed care plans, and federal- and state-level policies. Elucidating barriers at the health system level may reveal opportunities for sustainable solutions.

**Methods:**

To understand barriers to ambulatory care access for patients with Medi-Cal (California’s Medicaid program) and identify improvement opportunities, we performed a qualitative study using semi-structured interviews of a referred sample of clinicians and administrative staff members experienced with clinical patient encounters and/or completion of referral processes for patients with Medi-Cal (*n* = 19) at a large academic medical center. The interview guide covered the four process steps to accessing care within the health system: (1) scheduling, (2) referral and authorization, (3) contracting, and (4) the clinical encounter. We transcribed and inductively coded the interviews, then organized themes across the four steps to identify perceptions of barriers to access and improvement opportunities for ambulatory care for patients with Medi-Cal.

**Results:**

Clinicians and administrative staff members at a large academic medical center revealed barriers to ambulatory care access for Medi-Cal insured patients, including lack of awareness of system-level policy, complexities surrounding insurance contracting, limited resources for social support, and poor dissemination of information to patients. Particularly, interviews revealed how managed Medi-Cal impacts academic health systems through additional time and effort by frontline staff to facilitate patient access compared to fee-for-service Medi-Cal. Interviewees reported that this resulted in patient care delays, suboptimal care coordination, and care fragmentation.

**Conclusions:**

Our findings highlight gaps in system-level policy, inconsistencies in pursuing insurance authorizations, limited resources for scheduling and social work support, and poor dissemination of information to and between providers and patients, which limit access to care at an academic medical center for Medi-Cal insured patients. Many interviewees additionally shared the moral injury that they experienced as they witnessed patient care delays in the absence of system-level structures to address these barriers. Reform at the state, insurance organization, and institutional levels is necessary to form solutions within Medi-Cal innovation efforts.

**Supplementary Information:**

The online version contains supplementary material available at 10.1186/s12939-024-02174-8.

## Background

While insurance is integral for accessing healthcare in the US, coverage alone may not ensure access [1–5]. Individuals with Medicaid insurance experience more limited access to primary and specialty care, more fragmented care when receiving treatment, and more socioeconomic barriers compared to privately insured patients [6–12]. To promote equitable access to healthcare and mitigate racial and ethnic health disparities highlighted by the COVID-19 pandemic, institutions and policymakers must address challenges faced by Medicaid beneficiaries, who are disproportionally from minoritized communities [13, 14].

Medicaid is the largest US health insurance program by enrollment, and the majority are enrolled in managed care plans, where the state pays a fee for a managed healthcare plan to administer care [15, 16]. The transition away from fee-for-service (FFS) Medicaid, where the state directly pays providers for rendered services, to managed care, began in the 1970s and accelerated with the Affordable Care Act in 2010 [17]. Despite its aim to control costs, provide more predictable state spending, and improve quality, managed Medicaid has faced challenges recruiting specialist participation and added administrative complexity compared to FFS Medicaid [18–20].

In California, more than one in three Californians rely on Medi-Cal for insurance, and 80% of Medi-Cal (the state’s Medicaid program) beneficiaries participate in managed care plans [21, 22]. These managed Medi-Cal plans (MMC) are administered by various practice groups and local health systems who contract with health insurance organizations. In this context, we sought to systematically describe the range and impact of prevalent barriers to access for beneficiaries insured with Medicaid. We did this by interviewing clinical and administrative staff caring for patients with Medi-Cal seeking ambulatory primary and specialty care at an academic medical center. We describe challenges they experienced or witnessed on behalf of patients across four steps to accessing care: (1) scheduling, (2) referral and authorization, (3) contracting, and (4) the clinical encounter, as well as potential solutions to address them.

## Methods

### Context and setting

To identify opportunities to improve ambulatory access, we conducted a qualitative study at an academic medical center with over 60 primary and 180 specialty care sites primarily in Los Angeles County. We interviewed experienced, patient-facing clinical and administrative staff, henceforth referred to as “frontline staff,” about prevalent access challenges they recognized caring for ambulatory patients across the burden of illness spectrum from preventive to quaternary care.

This study’s academic medical center is a care-delivery contractor for a subgroup of MMC plans. Aligned with institutional goals to promote health equity, in November 2021 this health system initiated a Medi-Cal Ambulatory Access Task Force (hereafter, “Task Force”) to identify health system-specific barriers that impair patients with Medi-Cal from accessing ambulatory services and develop improvement recommendations. This qualitative study followed initial conceptual work by the Task Force, which developed a process map for patients with Medi-Cal accessing ambulatory care. Findings of this study were ultimately included in reports to academic medical center leadership for quality improvement. We used the Consolidated Criteria for Reporting Qualitative Health Research to report this study [23].

### Sampling and recruitment

To recruit a diverse sample of frontline staff including patient navigators, social workers, care coordinators, clinic directors, case managers, practice managers, financial counselors, physicians, and patient service representatives as potential interviewees, we began by requesting referrals from a lead social worker and physician from the Task Force. We first identified frontline staff that have frequent, regular interaction with patients with Medi-Cal insurance, including individuals experienced with clinical patient encounters (e.g., clinicians) and/or completion of referral processes (e.g., care coordinators) across ambulatory primary and specialty care. Using this snowball sampling approach, we asked these individuals to identify others in similar roles. We used email to invite this additional group to participate in our interview protocol (see Text, Additional File 1, which displays the introductory email invitation). This quality improvement study was exempted by the university’s Institutional Review Board.

### Interview guide

**Fig. 1:**
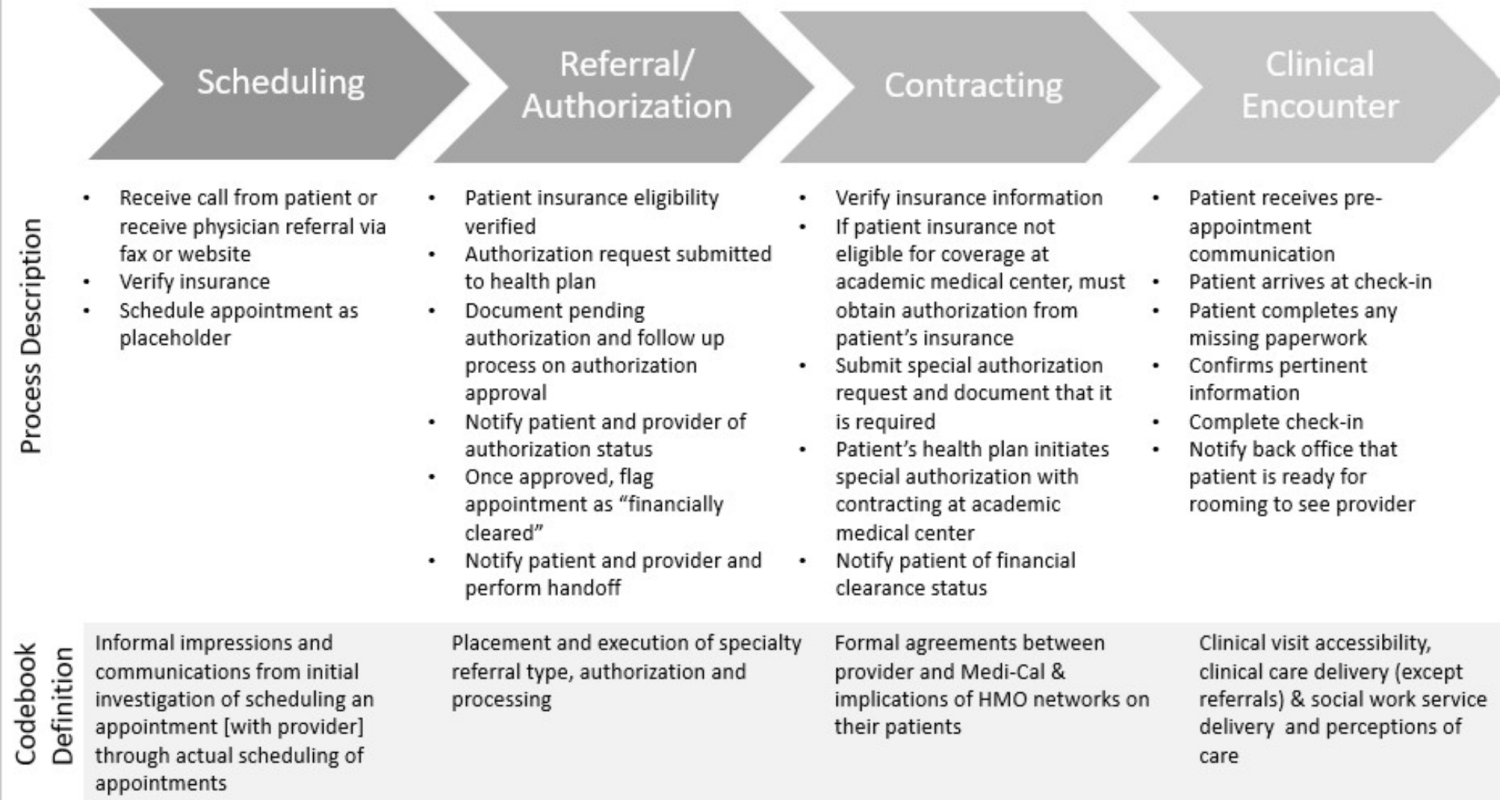
Taskforce-developed process map for patients with Medi-Cal insurance accessing ambulatory care at the academic medical center. Intake begins through physician referral or patient call to the patient communication center (PCC). This prompts the PCC to schedule an appointment that is reviewed by the financial clearance unit (FCU). If the patient’s health plan is not contracted with the academic medical center, the health plan must initiate a special authorization with the contracting department. Once the appointment has been reviewed by the FCU– and if special authorization is indicated, after the special authorization has been granted– the FCU notifies the patient and provider and the patient is cleared to attend their scheduled appointment.

The Task Force created a patient-flow process map for Medi-Cal insured patients, which included four key steps: scheduling, referral and authorization, contracting, and the clinical encounter (Fig. 1). We designed a semi-structured interview guide based on these steps to understand how well the process map described the actual experiences of interviewees.

Interviewees were asked to describe the flow of patient processes, what worked and did not at each step, and what process changes, if any, they would like implemented (see Text, Additional Files 2 and 3, which display the interview guides). Physicians were additionally asked about clinical care and perceptions of timeliness. The final section of the guide elicited potential solutions.

### Data collection procedures

The interview team included physician research fellows (M.B. and J.F.) and a fourth-year medical student (V.N.) previously trained in qualitative interviewing, as well as one medical resident (D.G.) with prior extensive qualitative research experience, and two medical residents who shadowed us (D.S. and K.T.). At least one member formally trained in qualitative interviewing (M.B., J.F., or V.N.) was present at each interview. Researchers conducted interviews between March and July 2022 via Zoom in a private setting. Interviewers informed interviewees of the project’s aims, confidentiality requirements, the voluntary nature of the interviewee role, and obtained consent for the interview and audio recording. Two interviewers participated in each interview with one conducting the interview and another taking detailed notes, which included reflections on interview content. The research team proofread and de-identified audio recordings transcribed using Otter.ai software.

### Analysis procedures

Team members (M.B, J.F, V.N) specified operational definitions of the four process steps for a priori codes (see Additional File 4, which details the process step definitions) allowing the interview team, within each step, to identify interviewee references to interventions at the state, health insurance organization, and institution levels. We used Microsoft Excel to construct matrices for analysis [24, 25]. In pairs, team members reviewed transcripts and coded text into one of the process step codes. The interview team discussed texts that did not align with one of the four process steps and created new inductive codes (for example, “solutions”). Finally, we reviewed the coded text for each process step and synthesized common patterns in the interviewee responses, which we describe as key “challenges.” The interview team met biweekly to debrief the interviews using the interview guide to review early findings and potential codes, and later to develop and review methods during the transcript coding process to confirm consistency in the application of coding across interviews. Interviews were conducted until thematic saturation was achieved [26].

## Results

Of 31 invited participants, 19 agreed to interview (Table 1**)**. Interviews ranged from 25 to 50 minutes, with a mean of 37 minutes (SD = 7). The most prevalent expressed reason for identified clinicians and staff not completing the survey was time constraints. Below we summarize results of the qualitative analyses of interview transcripts for each of the four process steps that guided our analyses. Each section is accompanied by a table (Tables 2 and 3) including quotes that illustrate challenges interviewees experienced caring for Medi-Cal insured patients.

**Table 1:** Participant characteristics^a^

	Participants (*n* = 19)
Role	
Administrator	7
Physician	6
Nurse	4
Social Worker	2
**Discipline**	
Primary Care, Adult	12
Surgical Specialty	5
Primary Care, Pediatric	1
Medicine Specialty	1

^a^The study team requested referrals from a lead social worker and physician from the Task Force. We first identified frontline staff that have frequent, regular interaction with patients with Medi-Cal insurance, including individuals experienced with clinical patient and/or completion of referral processes in primary care, surgery, and subspecialty clinics. We used the snowball sampling approach to invite these individuals to identify others in similar roles. We recruited these additional participants via email.

**Table 2:** Scheduling, referral and authorization: challenges and sample quotations

**A. Specialty clinics vary in acceptance of Medi-Cal insurance**
“There’s certain days doctors are taking Medi-Cal and certain doctors that don’t, that they’re not contracted. And so we just had to schedule an appointment with the doctors that did take Medi-Cal.” *(Administrator; Primary care)*
“But then when we would call again, they will tell us the same thing that, you know, these doctors weren’t available for like three, four months. And so that’s not ideal for someone that needs to get in right now. So it was just a lot of back and forth… it took almost a month to try to figure out who was going to see this patient or not.” *(Administrator; Adult primary care)*
**B. Authorization complexity leads to suboptimal and fragmented care**
“I’m sure it would happen at least once a week, if not more…it is a very sad and frustrating thing to witness, having the patient check in just to find out when they run the insurance, that it is not cleared.” *(Adult primary care administrator)*
“But a lot of those things [surgeries] can be done in a week or two as an outpatient. Which, if you have insurance, great, you can get back in easily and get that done. But if you have Medi- Cal, it’s a lot more challenging.” *(Surgical subspecialty nurse)*
“She was seen by urgent care and there was concern for like retinal detachment, and so she had an appointment to be seen, and they were going to even potentially do a procedure and the referral hadn’t been processed….on our side, the [patient services representative] said that they kept reaching out and reaching out to [the health insurance organization], and they hadn’t heard back. And so, you know, this is something that I was really concerned that was gonna hold up her procedure for this retinal detachment that was so time sensitive.” *(Adult primary care physician)*
“[The patient] has to go make an appointment in their system to see the doctor and get the referral… they can’t get an appointment for six weeks… you know, all these Medi-Cal HMO systems are terrible. The wait times are terrible.” *(Surgical specialty physician)*
“Getting stuff done is challenging with Medi-Cal, in terms of if I want to order a PET scan or radiation. Everything just takes longer…they have complete access just like anybody else, it’s just everything’s more of a pain.” *(Surgical specialty physician)*
“I understand there’s going to be a one-month delay, because we have to fax things and wait to hear back from [the health insurance organization], Medi-Cal. But for cancer surgery, there should be a fast track. I mean, maybe there is and I’m not aware. But I have that anxiety about the cancer patients. And for that reason, I will do my best to get them taken care of inpatient.” *(Surgical specialty physician)*
“We can only see certain patients when they’re hospitalized, and then they can no longer follow up at [the academic medical center] as outpatient…that really fragmented not only their specialty care, but also their primary care.” *(Adult primary care physician)*
“I think he’s been in an emergency room, not always ours, probably three or four times in the past year with his problem. So I know he is able to access care when he is having a problem. So I don’t think it’s going to delay his care in any way that affects his clinical outcome.” *(Surgical specialty physician)*
**C. Patients left confused by frequent Medi-Cal coverage changes and limited communication**
“Medi-Cal HMO doesn’t make it easy for the patient… What it would even take to process a referral for an HMO patient…you can times that by, you know, ten, and it’s that level of difficulty to try to process something for a Medi-Cal HMO patient.”*(Adult primary care administrator)*
“So we try to handhold and try to guide them in the right door. But a lot of times that just falls through the cracks, and there’s nothing we can do.” *(Adult primary care administrator)*
“The vast majority [of patients] have a difficult time, even understanding how the healthcare system works in general […] I find we’re doing a lot of education on that.”* (Adult primary care social worker)*
“We scheduled the patient and then the financial clearance unit says, ‘Oh no! This patient’s Medi-Cal expired on such and such day. Now they have this [different MMC health insurance organization] that needs like an authorization. Okay, fine… call the patient [to advise them that] we were just informed that your Medi-Cal expired on such and such date.’ We then call the patient to ask: ‘Do you know what [insurance you now] have?’ The patient responds, ‘No, you know, I wasn’t aware of that.’ So, the patient gets upset, jumped through hoops to get back on their Medi-Cal… [the authorization process] just prolongs, just delays the patient from being seen.” *(Surgical subspecialty administrator)*

*Abbreviations:* HMO, health maintenance organization; MMC, managed Medi-Cal; PCP, primary care physician

**Table 3:** Contracting and clinical encounter: challenges and sample quotations

**A. Non-medical support services infrequently accept Medi-Cal and have perceived lower quality**
“If we do have a patient who has Medicare A and B, which is their ideal insurance and pays out the most, we’ll tell them, hey, ‘I have this Medicare patient. But can you also see this Medi-Cal patient?’ in order to persuade the home health agency to take the Medi-Cal patient in a “bundle deal.” *(Adult primary care administrator)*
**B. Physicians’ understanding of and responsibility for insurance-related policies varied**
“Is it really the physician’s responsibility? Or is it the system’s responsibility? If we’re practicing at a tertiary care system… you could probably make a good argument it’s a system responsibility.” *(Surgical specialty physician)*
**C. Frontline staff need more assistance with patient care coordination needs**
“I am the only outpatient financial counselor for the facility… I am the only one within the facility [name redacted], that I’m aware of, to my knowledge, I haven’t been given any updates or memos indicating that there are other outpatient financial counselors…So I’m a one woman show.” *(Medicine specialty administrator)*
“I do a little bit more of this than I probably should or need to, but I do feel like it’s important to give the patient at the point of care, the instructions that they need to move forward with the care plan, I think it’s less ideal to say like, oh, ‘somebody will call you or somebody will message you.’ ” *(Adult primary care physician)*
“My office doesn’t have a social worker. I mean, I’m not going to offer [patients insured with Medi-Cal] any help with home health needs when they come to my clinic.” *(Physician)*
**D. Robust interpreter services benefit the Medi-Cal population**
“…patients don’t always speak the language. And so they’re very confused or overwhelmed. They’re extremely scared, because, they’re afraid to make a decision that may compromise being able to continue to access care…” *(Medicine specialty administrator)*
“…we communicate all of their appointment information directly through MyChart [patient portal for the electronic health record], so we encourage them to become familiar with it… The nice thing is that MyChart does have multiple language operators that can assist the patient, should the patient speak another language so that the patient can maneuver through MyChart.” *(Medicine specialty administrator)*
**E. Transportation barriers burden patients and families**
“$16 a day now for just one visitor parking…I mean, just for an hour is a lot.” *(Surgical specialty administrator)*
“Parking is really expensive, but I think a lot of them either use public transportation, although many have a family member drop them off. And that family member is driving around for an hour while the patient finishes their appointment…Sometimes family drops off the patient, and they leave and we don’t know what time they’re going to come back…” *(Adult primary care administrator)*

### Scheduling

Interviewees mentioned several scheduling barriers including perceived delays related to lack of timely financial clearance, difficulty accessing appointment slots for patients with managed Medi-Cal (MMC) compared with other insurance types, and specialty clinic provider hesitancy to schedule Medi-Cal insured patients. They noted variation in specialty medical and surgical clinics’ willingness to accept patients with Medi-Cal insurance. Interviewees described uncertainty about whether restrictions to access represented official health system policy, provider preference, or restrictions from Medi-Cal. Interviewees shared their impressions that difficulty scheduling appointments in sub-specialty surgery clinics reflected lower provider reimbursement for services to patients insured with Medi-Cal than for patients with other insurance coverage [27]. Interviewees also noted instances of referrals to sub-specialty clinics that were later declined by the MMC health insurance organization. These uncertainties and inconsistencies trickle down to the patient, with one interviewee stating, “This leaves patients confused, stating ‘I don’t understand why I can’t be seen by this doctor. I don’t understand what’s happening. Who am I supposed to go to?'”

### Referral/Authorization

All patients with MMC require referral authorization for visits with specialty providers. This is a complex process since MMC is an entity with variable networks for participating health insurance organizations. As each organization separately negotiates rates for their beneficiaries, administrators and patients find these heterogeneous processes confusing.

#### Authorization complexity seen as contributing to suboptimal and fragmented care

The academic medical center financially clears visits with MMC insurance organizations before a forthcoming appointment. If requirements for referral authorization have not been met (e.g. missing forms, lack of approval by MMC insurance organization), appointments had to be rescheduled, often resulting in clinical delays even for time-sensitive visits. For example, one interviewee expressed frustration regarding the inability to make timely post-operative outpatient appointments due to insurance coverage, stating, “I’m upset because we can’t reschedule the patients for like, a wound check…like an abscess that needs to be checked on within a week.”

Several physicians reported wait times for securing subspecialty appointments were longer for patients with MMC than for those with FFS Medi-Cal or private insurance. Delays were attributed to the processes required for MMC insurance organizations to authorize care. This extended to imaging studies, as patients with MMC were constrained to certain imaging centers outside the academic medical center. Surgeons cited some instances where external images were lower quality, resulting in added pre-operative coordination and surgical delays.

Hospitalized MMC-insured patients were considered particularly vulnerable to care disruptions during transitions to outpatient care. Post-discharge referrals were often delayed due to lengthy authorization processes by MMC insurance organizations. To avoid delays in care, providers reported sometimes performing otherwise outpatient workups during the hospitalization, possibly increasing the length of stay and costs of inpatient care.

#### Patients were often confused by frequent coverage changes and limited communication

Interviewees reported patients varied in their familiarity navigating MMC insurance. Patients were usually unaware of monthly changes in Medi-Cal coverage or MMC insurance organization assignments. This frequently produced emotional stress for patients. Furthermore, if gaps in insurance knowledge were not addressed, patients remained at risk for adverse clinical outcomes. Interviewees also reported that MMC does not consistently notify ordering providers of declined authorizations, which contributed to provider frustration and poor communication between patients and providers.

### Contracting

Authorization requirements for patients’ ambulatory academic medical center visits varied according to existing contracts between the medical center and MMC insurance organizations.

This complexity made it difficult for frontline staff to remain current with contractual requirements for each patient’s specific plan. Interviewees recommended development and wide dissemination of Medi-Cal policy education.

#### Access to ancillary support services perceived as limited and lower quality

Interviewees reported challenges in delivering comprehensive patient care due to limitations in support services offered by MMC contracts, with many outpatient physical and occupational therapy units, for example, not accepting Medi-Cal. Interviewees also reported challenges negotiating care with home health agencies and skilled nursing facilities for Medi-Cal insured patients. They reported that arranging support services was especially time-consuming, unless patients had concomitant Medicare insurance. Even then, the quality and specificity of support services available for patients insured with Medi-Cal raised concerns. This is illustrated by one interviewee who shared, “Ultimately, for those Medi-Cal patients that are very, very sick, we try and make sure that they have Medicare, as well, because they’ll get better care and be accepted into a lot of different facilities. If they just have Medi-Cal… Those facilities are pretty sad.”

#### Physicians’ understanding of and responsibility for insurance-related policies varied

While overall, physician interviewees stated that insurance type does not affect the clinical care they provide, physicians varied substantially in their understanding of, commitment to, and receipt of support for understanding insurance-related complexities. While most wanted to know which specialty providers were available to see patients with specific MMC plans, many expressed the view that others within the health system should manage tasks associated with insurance complexities. Physician interviewees, frustrated by repeated denials or lack of closure with previously requested referrals for their patients, described wide variations in their efforts to place referrals for Medi-Cal insured patients.

### Clinical encounter

Interviewees acknowledged patients’ appreciation for receiving comprehensive care at the academic medical center, but described patient and provider frustration when access to clinical care was delayed, abridged, or inadequately supported.

#### Frontline staff reported needing more assistance for patient care coordination

Interviewees depicted the important need for care coordination, specifically for complex patients with Medi-Cal who require frequent follow-up visits and support at home [28–30]. They also noted concerns about the adequacy of time allocated for them to provide sufficient care coordination while also managing large caseloads. For example, one social worker covers high-risk patients in twelve clinics with limited support from financial counselors. Very often, individual frontline staff members are the agents primarily responsible for both patient and family education in addition to care coordination. While interviewees are highly dedicated to their work, they noted how time-consuming the tasks can be, often exceeding their job scope.

#### Interpreter services seen as essential for the Medi-Cal population

Interviewees noted that Medi-Cal patients were more likely to have limited English proficiency than other patients and that patients with limited English proficiency experienced unique communication barriers that sometimes compromised care [31–33]. While telephone and video visit interpreters were routinely used in ambulatory clinics, interviewees noted that interpreter use often lengthened visit times [34, 35]. Bilingual frontline staff were deemed invaluable resources.

#### Transportation-related barriers burdened patients and families

One interviewee noted that MMC offered good transportation resources, specifically for elderly patients. However, parking remained a frequently noted obstacle for those who drive, and many interviewees reported high parking costs. Parking barriers also affect family members, who missed accompanying the patient to the appointment since they continue to drive during the visit to avoid parking costs. This inconvenience was highlighted by one interviewee who stated, “We used to have patients that would be dropped off at 8 am for an appointment. And I would be walking out at 5:00/5:30 pm. And they were still there because their transportation hadn’t arrived.”

### Proposed interviewee solutions

Recommendations by interviewees aimed to educate staff and providers, increase the number and consistency of contracts between the academic medical center and Medi-Cal, and expedite authorization processes to decrease confusion and delays. The majority of suggestions applied to staff and provider education. Suggested modes of education included special training for frontline staff to more efficiently navigate Medi-Cal related processes, disseminating updates to staff directly from state administrators of Medi-Cal, and crafting a “guide to insurance” for providers.

Interviewees frequently referenced their observations from neighboring county hospitals to provide examples for how to address the needs of Medi-Cal insured patients. For example, one suggested that the consolidation of ancillary services such as durable medical equipment, occupational health, and physical therapy in one physical location at the health center would be helpful for Medi-Cal insured patients. Other suggested solutions included additional support staff with expertise in Medi-Cal insurance processes to assist with troubleshooting insurance-related issues in clinic, and access to a pharmacy program for assistance filling medications and identifying the most affordable formulary options. More globally, providers recommended increased expansion of specialty clinic locations to underserved areas, with the goal of meeting a metric of caring for a certain percentage of Medi-Cal insured patients.

## Discussion

Our study explored perspectives of frontline staff caring for patients insured with Medi-Cal in a large academic health system. We interviewed clinical and administrative staff experienced caring for patients with Medi-Cal to learn about barriers to accessing care associated with scheduling, referral authorization, contracting, and the clinical encounter. Our findings highlight gaps in system-level policy, inconsistencies in pursuing insurance authorizations, limited resources for scheduling and social work support, and poor dissemination of information to and between providers and patients. Many interviewees shared the moral injury that they experienced as they witnessed patient care delays in the absence of system-level structures to address barriers [36–38]. Overall, we found a need for health system process improvements and larger scale policy solutions within Medi-Cal innovation efforts.

Difficulty accessing timely ambulatory care for Medicaid-insured patients is well-documented in the literature [29, 30]. Our interviews revealed how MMC impacts academic health systems through additional time and effort by frontline staff to facilitate patient access compared to FFS Medicaid. Interviewees revealed that staff caring for patients with Medi-Cal often rely upon self-education, time-consuming phone calls, language skills other than English, and novel workarounds to navigate challenges to accessing even routine aspects of care. Staff develop specialized knowledge that allows them to navigate a complex, heterogeneous system on behalf of patients who often present with urgent, and/or chronic needs. Additionally, they act as intermediaries between patients, their families, and multiple internal and external players, including the hospital system, managed care plans, and California’s Medicaid program.

### Proposed author solutions

Most existing literature focuses on state-level access issues for Medicaid-insured patients, while few describe health system-level barriers or solutions to improving access [8–10, 39–42]. In our study, interviewees prioritized education of frontline staff, increases in the number and consistency of health system contracts with Medicaid, and standardization of authorization processes to address reported barriers to care. While these strategies are likely to improve patient access, implementation of these interviewee-led solutions will require integration with evolving state Medicaid policies, health insurance organizations, and health systems. Our discussion below provides a framework for melding systemic Medi-Cal challenges with the priorities of frontline staff, and proposes the following multi-level solutions for improving access to care for Medi-Cal insured patients.

#### State-level

Managed Medi-Cal aims to promote care quality and access through mechanisms such as a directed payment options, through which states allow MMC organizations to directly pay network providers, with the hopes of improving provider participation and encouraging value-based practices [43]. Evaluation of the effects of these payment mechanisms on care access across states vary, and our study illustrates persistent challenges despite their implementation in California. In 2018, the California Advancing and Innovating Medi-Cal (CalAIM) initiative was established by the Department of Health Care Services to optimize the Medi-Cal beneficiary experience by addressing social drivers of health, standardizing systems to reducing complexity, and improving outcomes through payment reform [44]. Our interviewees candidly reported that low Medi-Cal reimbursement limits physician participation in the care for these patients. Matching appropriate financial reimbursement to the needs of the Medi-Cal population is paramount. In 2023, the California state budget was revised to increase Medi-Cal provider rates for primary care, maternity care, and non-specialty mental health services [45, 46]. If the state were to adjust Medi-Cal funding for outpatient care to align with funding available for inpatient care, [47]health systems could be incentivized to expand access to ambulatory care, thus promoting overall cost-savings by preventing inpatient hospitalizations [48–51]. Reimbursement incentives addressing social drivers of health could also be leveraged to support Medi-Cal insured patients [52, 53].

#### Health Insurance Organization-Level

Under managed Medi-Cal, health insurance organizations assume an enormous role as they are responsible for approving referrals and authorizing patients to receive care within or outside of their network. While interviewees frequently expressed frustration about delayed and rejected referrals, they rarely acknowledged the key roles that medical groups and independent physician associations (IPAs) played in making these decisions. Removal of these intermediaries from making decisions about referral authorizations may broaden access and decrease barriers, as suggested with the recently approved direct Medi-Cal contract with Kaiser Permanente [54]. Additionally, the standardization of Medi-Cal enrollment processes and benefits through CalAIM may mitigate contracting complexities.

Given interviewees shared that insurance-related delays resulted in progression of disease for Medi-Cal insured patients, health insurance organizations must consider cost savings to the broader health system for providing comprehensive care for this high-need population. This can be achieved by partnership between Medi-Cal and specific health systems to harness health system strengths (e.g. specialty care) and ensure patients have meaningful access. This requires robust data collection regarding this patient population’s needs and care utilization, along with accountability for patient outcomes [55]. Metrics such as time from referral to appointment and referral completion rates can be used to assess equity between Medicaid and other insurance plans [56].

#### Institution-Level

To optimize the alignment between institutional policies and frontline experiences, we propose that health systems evaluate their own Medicaid ambulatory care barriers and include insights from their frontline staff in the assessment [56]. Staff training by content experts and/or primers on state-level insurance practices paired with technological advances to streamline authorization processes can increase clinician and staff understanding of Medicaid plans and reduce administrative delays [57]. Academic health centers, especially, have a responsibility to streamline these processes, as community hospitals and remote health centers that house Medi-Cal insured patients need to refer them to specialty care only offered at these academic health centers. Efforts to standardize and complete financial clearance prior to scheduled appointment times could reduce delays in appointment access, diagnoses, and treatment. These strategies can be especially helpful for those who may face additional language barriers and/or live farther geographically. To better understand the impact of social drivers of health on this population, health systems need to build capacity to screen and address social drivers of health, as is now required by regulatory bodies, including the Center for Medicare and Medicaid Services [58, 59].

We began this work in our health system by educating clinical departments on health system acceptance of Medi-Cal patients and training call center staff to standardize management of Medi-Cal scheduling and financial clearance. Next, we are interviewing patients with Medi-Cal to better understand their care preferences and priorities. Engaging patients provides the opportunity to incorporate community needs and increase participation among an often-ignored population [60].

### Limitations

This study has several limitations. Snowball sampling of interviewees may have resulted in sampling bias, although the identification of frontline staff with more familiarity working with patients with Medi-Cal helped to provide more specific areas of challenges and solutions. Interviewers were physicians with preexisting relationships with some interviewees, which may have positively biased discussion. However, their collegial relationships may have also fostered openness for interviewees to raise areas of improvement. Recall bias may have impacted our results, however most of the themes were reported by several interviewees in our sample. We forewent pilot testing of the interview guide given the expeditious timeline of the Task Force [61]. We described frontline staff perspectives in one academic medical center; staff working in other health systems may report different experiences. Finally, while mental healthcare is crucial for the Medi-Cal population, behavioral health in LA County has different regulations than medical care, thus was not discussed here.

## Conclusion

This study revealed barriers to ambulatory care access for Medi-Cal insured patients identified by frontline staff. These barriers included lack of awareness by interviewees of system-level policy, complexities surrounding insurance contracting, limited resources for social support, and poor dissemination of information to patients. Interviewees perceived that these logistical barriers resulted in care delays and fragmentation that negatively impacted patients. This work highlights a need to promote both innovative larger scale policy solutions, including reimbursement reform, and health system process improvements to provide equitable care for Medi-Cal insured patients.

### Electronic supplementary material

Below is the link to the electronic supplementary material.

Additional File 1: Introductory email invitation to potential participants.

Additional File 2: Semi-structured interview guide, non-physician participants.

Additional File 3: Semi-structured interview guide, physician participants.

Additional File 4: Process step definitions as a priori codes.

## Data Availability

No datasets were generated or analysed during the current study.
