# Supplementary material for: Frontline perspectives on barriers to care for patients with California Medicaid: a qualitative study
Source: Int J Equity Health. 2024 May 22;23:102. doi: 10.1186/s12939-024-02174-8 (PMC11110184; doi:10.1186/s12939-024-02174-8)
Supplement: Supplementary file 1 — Additional File 1: Introductory email invitation to potential participants. [file 12939_2024_2174_MOESM1_ESM.docx]

**Additional File 1:** Introductory email invitation to potential participants

Dear XXX,

I am one of the [academic medical center (AMC)] fellows helping with an initiative to better understand the system level barriers our Medi-Cal patients face in getting outpatient care at [AMC].  XXX mentioned your name as someone who could illustrate how the patients at various [AMC] primary care clinics who have Medi-Cal fare going through our system, and what some of the challenges are in getting them into follow up care.  I would appreciate hearing your insights or reflections from your experiences and highlight these gaps to the administration, ultimately with the hope of improving these processes for our patients.

Would you be willing to have a <30min phone or zoom conversation on this topic in the next 1-2 weeks? [Name], cc’d, is my co-fellow working on this project with me.

If you do not work with Medi-Cal insured patients, are unfamiliar or not comfortable discussing this topic, feel free to decline or disregard this e-mail.

Thanks for considering and look forward to hearing from you.
