# Supplementary material for: Frontline perspectives on barriers to care for patients with California Medicaid: a qualitative study
Source: Int J Equity Health. 2024 May 22;23:102. doi: 10.1186/s12939-024-02174-8 (PMC11110184; doi:10.1186/s12939-024-02174-8)
Supplement: Supplementary file 3 — Additional File 3: Semi-structured interview guide, physician participants. [file 12939_2024_2174_MOESM3_ESM.docx]

**Additional File 3:** Semi-structured interview guide, physician participants

**INTRODUCTION:**

For our project we are interested in your experiences in facilitating care for patients with Medi-Cal insurance at [AMC]. The purpose is to identify areas where the health system can improve common issues that arise, from getting referrals all the way through scheduling and billing as part of a quality improvement initiative. Your participation in this conversation is voluntary with no penalty if you decline or choose not to answer any of the questions. If you agree to participate we will ask you to describe the ways you interact with the needs of Medi-Cal patients, your observations on barriers to having clinical visits at [AMC], and any suggestions you may have. It will take 20-30 minutes.

Your identity and personal information will not be disclosed, and our notes will not have your name attached. You may contact us if you have any questions.

Do you have any questions or would it be okay to begin? To help us capture all your important stories, do you mind if we audio record our conversation?

**QUESTIONS:**

**Information about interviewee & general insurance knowledge:**

1. We’d like to hear about your role within our health system since you see how things really happen behind the scenes. What does your job typically entail?
   1. *Optional follow up:* What are the factors that drew you to this work?
2. Tell me what you know about patient insurance coverage and how it works?
   1. *Probe:* how do you find out?
3. Tell me what you understand about the referral process itself?
   1. *Probe:* In what cases do you use the EMR or other means to find out the patient’s insurance?

**Interviewee Medi-Cal knowledge and experience:**

1. How often and how deeply do you work with people with Medi-Cal insurance?
2. Tell me about your understanding of whether patients with Medi-Cal insurance are able to establish [primary and specialty] care at [AMC]?

**Clinic & system level (contracting, referrals & authorization):** For the next several questions, we’re really interested in hearing about the barriers and challenges you face in helping patients with Medi-Cal insurance get care at [AMC].

1. Think about the last patient with Medi-Cal you saw in your clinic or referred them to a specialist. Were there any hurdles with the referral or authorization in process?
   1. *Follow up:* Have these issues led to any delays in care, and if so, how did they impact the clinical outcomes?
   2. *Follow up:* How does this look different for patients with Medi-Cal fee-for-service versus managed care or HMO plans?
   3. *Follow up:* Do you have resources or certain people to go to if you have questions on how to handle problems that arise for your patients with Medi-Cal?
      1. *Alternative:* How have you gained knowledge of how Medi-Cal works? Do you feel equipped to handle insurance issues that arise?
   4. *Follow up:* In the last 6 months, was there a time where you were told by a [AMC] clinic that they would not take a patient due to their Medi-Cal insurance?
      1. *If yes:* What type of Medi-Cal did the patient have
         1. What were the reasons given for this?
         2. How do you think the patient impacted?
2. What roadblocks do you commonly face **internally** within the systems at [AMC] in getting care for Medi-Cal patients?
   1. *Follow up:* What **external** roadblocks do you encounter in getting care for Medi-Cal patients, such as with Medi-Cal or with the Medi-Cal HMO groups?
   2. How do these challenges impact your clinic workflow?
3. Do you ever have to use workarounds to get Medi-Cal patients into the specialists or other outpatient services they need?
   1. *If yes:* What does that look like?

**Provider & Patient level (clinical encounter, communication):** For the next several questions, I’d like you to think of the recent patients with Medi-Cal that you cared for. [*pause*].

1. Thinking about this patient, what went well during the clinic visit and what didn’t go well for that patient?
   1. *Follow up:*  Do you feel equipped to manage the full range of medical and social needs that come with these patients?
2. Considering the location of your work, what do patients tell you about how accessible the clinic is in terms of things like transportation and parking?
3. Do you notice any different challenges in your role for patients whose primary language is not English?
4. If you use MyChart to communicate with patients, what proportion of patients are using MyChart?
   1. *Follow up*: Do you notice any differences in MyChart use among patients with Medi-Cal compared to those with other types of insurance?
5. Any other challenges for patients come to mind from your vantage point?

How did they accrue Medi-Cal insurance knowledge?

How would you want to receive information and what gaps are most important?

Should we ask about no-shows or cancellations?

**Solutions and Insights:** Let’s shift gears and discuss how we could make things better.

1. Could you describe some ways you are proud of the care we provide for patients with Medi-Cal?
2. How could [AMC] evolve to better care for patients with Medi-Cal?
3. If you could tell the leaders of [AMC] anything, what would you tell them?
4. Anything else about your experience with Medi-Cal patients that you want to share?

What you shared today is very valuable and thank you for your time.
I will turn off the recording now.

You are welcome to contact us if you have any questions, and do you give us permission to reach back out to you for more conversation or idea generation?
