# Supplementary material for: Frontline perspectives on barriers to care for patients with California Medicaid: a qualitative study
Source: Int J Equity Health. 2024 May 22;23:102. doi: 10.1186/s12939-024-02174-8 (PMC11110184; doi:10.1186/s12939-024-02174-8)
Supplement: Supplementary file 4 — Additional File 4: Process step definitions as a priori codes. [file 12939_2024_2174_MOESM4_ESM.docx]

**Additional File 4:** Process step definitions as a priori codes

| **PROCESS STEP** | **DEFINITION** | **EXAMPLES** |
| --- | --- | --- |
| (1) Appointment scheduled | *Informal impressions and communications from initial investigation of scheduling an appointment at academic medical center (AMC) through actual scheduling of appointments.* | -Perceptions on reasons related to specific providers/clinics not taking Medi-Cal  -Wait times  -Trying to get patient into appt and being told  delay to see provider 2/2 availability  -Inpatient to outpatient transition -Patient call center (PCC) issues  -Discussions of sending patients to a county site |
| (2) Referral / Authorization | *Impressions and communication regarding placement and execution of specialty referral type, authorization and processing* | -Commentary on how easy/hard getting referrals authorized -Not being financially cleared in time for the scheduled appointment -Getting services approved  -Referral/authorization examples  -Paperwork/calling to follow up authorizations  -Time needed for patient service representative (PSR)'s or providers to explain processes to patient -Interviewees attributing issues to HMO network |
| (3) Contracting | *Formal agreements between AMC and Medi-Cal & implications of HMO networks on their patients (*Medi-Cal/HMO-assigned primary care)* | -Specific mention of lack of contract -Interviewee describing resources to discern what the contracted services are (e.g. by PSRs) -Education about contracted services -Hard to find PTs and behavioral health contracted to take Medi-Cal -Using existing forms to get approved care from Medi-Cal for additional / continued services at AMC -Communications from Medi-Cal/Plans to patients |
| (4) Patient Clinical Encounter | *Clinical visit accessibility, clinical care delivery (except referrals) & social work service delivery and perceptions of care* | -Accessibility: transportation issues, language barriers, MyChart challenges -Care provided by social workers -Social work direct interactions with patients -Front desk issues upon patient arrival -Quality or processes of clinical care (including documentation) -Real or perceived challenges for providing care to Medi-Cal patients (i.e. needing more time, more frequent visits) |
|  |  |  |
| **LEVELS** | **DEFINITION** | **EXAMPLES** |
| System-level: Academic Medical Center (AMC) | *Issues related to AMC system-wide policies and procedures, including EMR* | -Challenges due to AMC  -Instances of interviewee attributing problem to AMC -Perception of need for AMC-led communication (e.g.: patient tells staff "nobody told me the referral was denied") |
| System-level: Medi-Cal & its health plans | *Issues related to Medi-Cal and its health plans' policies and procedures* | -Perceived lack of communication from medical (re: PCP assignment, lack of patient knowledge of being in an MC HMO) -Wait times for paperwork turnaround  -Instances of people blaming Medi-Cal/state (e.g., lack of training) -Challenges due to Medi-Cal/state policy level issues |
| System-level: Clinic | *Clinic-specific variations on established policies/procedures/norms* | -De facto clinical policy that differs from health system policy  -Actual or perceived front desk policies -Clinic-specific workarounds |
| Staff level: Provider | *Provider level variations in workflow; provider viewpoints/perceptions* | -Commentary / perception that providers determine/block access -Instances of bias (e.g., perceptions that some providers take longer to see Medi-Cal patients)  -Provider lack of knowledge/education on insurance -Provider-specific workarounds  -Provider-to-provider communication (e.g. PCP to specialist) |
| Staff level: Staff | *Staff level variations in workflow; individual staff member's viewpoints/perceptions* | -Speaking the same language as their patients  -Personal beliefs / mission towards the Medi-Cal population  -Impacts of length of time worked at AMC/experience  -Time burden related to insurance work  -Staff-specific workarounds |
| Patient-level | *Comments related to patient needs, challenges, and their external environment. Comments related to patient-led navigation of system* | -Variation in patient ability/knowledge of navigating systems  -Patient support systems and its impact  -Community perceptions of AMC -Variation in ability to get prescribed treatments (medications and tests/services) |
